# Deep learning based automatic segmentation of the Internal Pudendal Artery in definitive radiotherapy treatment planning of localized prostate cancer

**DOI:** 10.1016/j.phro.2024.100577

**Published:** 2024-04-15

**Authors:** Anjali Balagopal, Michael Dohopolski, Young Suk Kwon, Steven Montalvo, Howard Morgan, Ti Bai, Dan Nguyen, Xiao Liang, Xinran Zhong, Mu-Han Lin, Neil Desai, Steve Jiang

**Affiliations:** Medical Artificial Intelligence and Automation (MAIA) Laboratory and Department of Radiation Oncology, University of Texas Southwestern Medical Center, Dallas, TX, USA

**Keywords:** Deep learning, Automatic Segmentation, Internal Pudendal Artery (IPA), Erectile Dysfunction, Prostate cancer

## Abstract

**Background and purpose:**

Radiation-induced erectile dysfunction (RiED) commonly affects prostate cancer patients, prompting clinical trials across institutions to explore dose-sparing to internal-pudendal-arteries (IPA) for preserving sexual potency. IPA, challenging to segment, isn't conventionally considered an organ-at-risk (OAR). This study proposes a deep learning (DL) auto-segmentation model for IPA, using Computed Tomography (CT) and Magnetic Resonance Imaging (MRI) or CT alone to accommodate varied clinical practices.

**Materials and methods:**

A total of 86 patients with CT and MRI images and noisy IPA labels were recruited in this study. We split the data into 42/14/30 for model training, testing, and a clinical observer study, respectively. There were three major innovations in this model: 1) we designed an architecture with squeeze-and-excite blocks and modality attention for effective feature extraction and production of accurate segmentation, 2) a novel loss function was used for training the model effectively with noisy labels, and 3) modality dropout strategy was used for making the model capable of segmentation in the absence of MRI.

**Results:**

Test dataset metrics were DSC 61.71 ± 7.7 %, ASD 2.5 ± .87 mm, and HD95 7.0 ± 2.3 mm. AI segmented contours showed dosimetric similarity to expert physician’s contours. Observer study indicated higher scores for AI contours (mean = 3.7) compared to inexperienced physicians’ contours (mean = 3.1). Inexperienced physicians improved scores to 3.7 when starting with AI contours.

**Conclusion:**

The proposed model achieved good quality IPA contours to improve uniformity of segmentation and to facilitate introduction of standardized IPA segmentation into clinical trials and practice.

## 1 Introduction

Treatment of localized prostate cancer (PCa) with radiotherapy (RT) is highly effective but incurs significant quality of life impact, of which erectile dysfunction (ED) is the most common long-term toxicity for initially potent men. Prior strategies to reduce ED after RT by dose-reduction to erectile tissue below the prostate [1] or with use of oral phosphodiesterase 5 inhibiors (i.e. RTOG 08-15) [2] have been unsuccessful.

Advancements in stereotactic ablative radiotherapy (SAbR) and MR-aided targeting in prostate cancer (PCa) have focused on sparing critical neurovascular structures near the prostate, especially the internal pudendal arteries (IPA), vital for penile blood supply. A trial [3] showed benefits of reducing dose to the IPA, leading our institution to conduct a multi-institutional study on individualized sparing of these structures. However, standardizing IPA segmentation is challenging, affecting study consistency. We used educational atlases and rapid review QA workflows, but their resource intensity limits wider use in practice and further research. There's a need for innovative tools to streamline learning and expedite QA processes.

Indeed, contouring inconsistencies are known but understudied in clinical radiation therapy trials. Previously, Thor et al., [4] used a coherent heart definition enabled through their open-source deep learning (DL) algorithm to evaluate heart doses in the RTOG 0617 trial. The trial heart doses were found to be significantly higher than previously reported, which they concluded may have led to an even higher mortality rate. Auto-segmentation is likely to reduce contouring and dose inconsistencies while increasing the quality of clinical RT trials and routine clinical treatments.

Automatic segmentation has drawn enormous attention in RT since it reduces the contouring time drastically and creates contours with less intra- and interobserver variability. Recently, convolutional neural networks (CNN) were adopted to advance structure delineation accuracy significantly [5], [6], [7], [8], [9], [10], [11], [12], [13], [14], [15], [16], [17], [18], [19], [20], [21], [22] Many groups have designed novel deep CNN architectures for pelvic organ segmentation [14], [15], [16], [17], [18], [19], [20], [21], [22] Several of these papers have focused specifically on preoperative prostate GTV and OARs [14], [15], [16], [17], [18], [19], [20], [21] and a few on post-operative prostate CTV and OARs [21], [22].

Automating IPA segmentation via AI faces multiple challenges: limited and noisy historical data for IPA segmentation, as it's not typically marked as an organ-at-risk, with considerable variation in contours, especially in the cranial-caudal direction and inconsistencies in contour start and end points. Additionally, IPA contours often include muscles or bones due to contouring tool limitations, further complicating the training dataset. The limited contrast in CT images means MRI fusion is necessary for accurate IPA contouring, but treatment planning systems align primarily with prostate segmentation fiducials, not ideal for IPA accuracy. Furthermore, not all patients have MRI available, requiring the AI model to work effectively with varied inputs, such as CT-alone scenarios.

In this work, we aimed to address all the above challenges and develop an auto-segmentation model for the IPA to reduce contouring inconsistences, to improve uniformity in IPA segmentation for clinical trials, and to facilitate the introduction of IPA for clinical planning. Allowing model training with a noisy data set is more than needed for future model maintenance when multiple institution data is included. A previous research work [23] has introduced and evaluated the use of Deep Learning (DL) models for the automated segmentation of IPAs on MRI. Our current study is pioneering as it marks the first instance of employing models for IPA segmentation on CT that has limited soft tissue contrast – deep learning or otherwise.

## 2 Materials & methods

### 2.1 Dataset

The model training and evaluation dataset consists of CT and T2 weighted MRI for 56 patients with prostate cancer treated at a large medical institution in the United States from 2017 to 2021. In this dataset, the IPA was segmented by multiple physicians and reviewed and revised by an expert physician. All CT images were acquired using a 16-slice CT scanner (Royal Philips Electronics, Eindhoven, The Netherlands), and these CT images were acquired with voxel size 1.17 mm × 1.17 mm × 2.5 mm. MRI images were acquired with a voxel size of 0.56 mm × 0.56 mm × 3 mm. The MRI was a rigidly registered CT with a fiducial-based registration most appropriate for prostate segmentation. Fourteen patients were used as the test dataset, and the remaining 42 patients were used for cross-validation. The IPA labels were cleaned to ensure uniformity in the extent of the structure to be segmented. The caudal structure limit was determined as 1 cm beyond the primary target (prostate) planning treatment volume (PTV).

An additional 30 patients treated at the same institution from 2020 to 2021 were recruited for two “inexperienced” physicians (no prior IPA segmentation training) to contour with and without the assistance of the AI model as part of the observer study, since this AI model was developed to assist physicians in contouring the IPA. The details of the observer study are described below.

### 2.2 Model

**Fig. 1:**
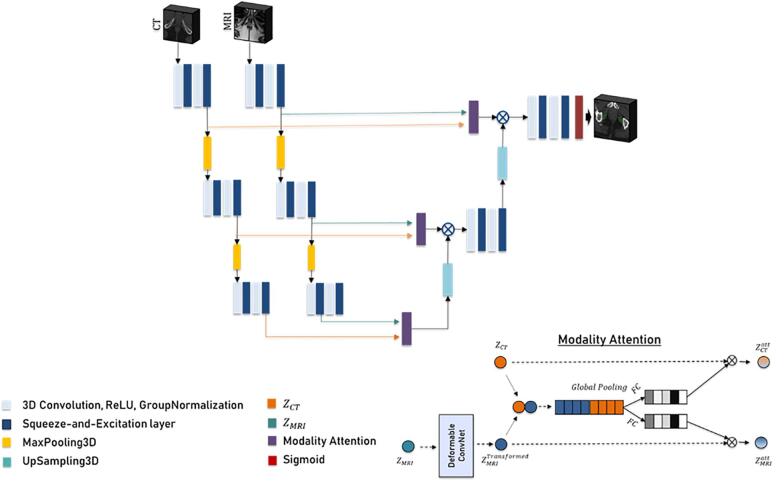
Proposed UNet architecture with modality attention and squeeze-and-excite block for IPA segmentation using CT and MRI or CT alone as input.

The model architecture has a UNet [24] backbone. The model was designed to satisfy three conditions: effective learning of small structures, learning appropriate and mutually exclusive features from MRI and CT, learning the effective flow between MRI and CT for an effective combination of features while avoiding additional rigid registration to bones, and to produce acceptable segmentation when MRI is unavailable. The model architecture proposed is shown in Fig. 1, and each component of the model is explained in detail below.

#### 2.2.1 Squeeze and excite blocks (SE) to improve feature loss in downsampling

Standard U-Net's multiple downsampling layers, while useful for high-level feature learning, can hinder segmentation of small structures like the IPA. To address this, we limited our network to two pooling layers and incorporated SE [25] blocks for better feature learning. The SE layer enhances the network's capacity for dynamic feature recalibration across channels. Additionally, we extended 2D squeeze, excitation, scale, and convolutional functions to 3D, enabling direct extraction of 3D features from CT images, thereby boosting the network's representational abilities.

#### 2.2.2 Modality attention to enhance feature learning from MRI and CT

To enable effective learning of features from MRI and CT, we used an effective modality attention block in the skip connection before concatenating them with the decoder layers. Separate encoders were used for extracting MRI and CT features. For designing an effective modality attention unit, several conditions need to be met: 1). It should be completely data-driven, 2). It should be able to perform attention effectively from two raw feature maps that are offset with each other, and 3). It should be light enough to avoid additional computational overhead. To satisfy all three conditions, it is necessary to consider feature-based self-attention instead of any attention mechanism that involves computationally complex dot products. We constructed modality attention using global average pooling and fully connected layers. The modality attention block consisted of a deformable convolution layer [26], which learns the offsets for the MRI necessary for effectively comparing MRI features with that of the CT. Normal convolution layers cannot model large unknown transformations because of their fixed geometric structure. The 3D deformable convolution layer adds a 3D offset to enable learning of a deformed sampling grid. These offsets are learned simultaneously along with convolution parameters.

The MRI and CT features go through the following steps in the modality attention module:

(1) $$Z_{\mathrm{MRI}}^{\mathrm{Tran}}={\mathrm{DefCon}(Z}_{\mathrm{MRI}})$$

(2) $$Z_{\mathrm{MRICT}}=Z_{\mathrm{CT}}+Z_{\mathrm{MRI}}^{\mathrm{Tran}}$$

(3) $$Z_{\mathrm{GP}}=\frac{1}{W\times H\times D}\sum_{i=1}^{W}\sum_{j=1}^{H}\sum_{k=1}^{D}Z_{\mathrm{MRICT}}\left(i,j,k\right)$$

(4) $$Z_{\mathrm{CT}}^{\mathrm{att}}=SoftMax\left(Z_{\mathrm{GP}}\right)\times Z_{\mathrm{CT}}$$

(5) $$Z_{\mathrm{MRI}}^{\mathrm{att}}=SoftMax\left(Z_{\mathrm{GP}}\right)\times Z_{\mathrm{MRI}}^{\mathrm{Tran}}$$

(6) $$Z_{\mathrm{encoder}}=Z_{\mathrm{CT}}^{\mathrm{att}}+Z_{\mathrm{MRI}}^{\mathrm{att}}$$

The final architecture of the model which is a **UNet** with **S**queeze and excite blocks and **M**odality **A**ttention will be referred to as SUNet-MA henceforth.

#### 2.2.3 Modality dropout (MoDO)

To ensure that the model can work well with only CT as input, we used MoDO where MRI is dropped out randomly from the input during training. This training strategy forces the network to use information from both modalities, even when one modality is so highly correlated with output that the other would otherwise largely be ignored. This also enables the model to be used in the absence of MRI as input.

#### 2.2.4 Loss functions to correct Bone/Muscles in IPA training data

The IPA's small size and limited contour resolution often result in its structures containing muscle and bone tissue, leading to noisy training data and reduced model performance. To address this, we employed an unsupervised method for label correction. As MRI provides key anatomical details for IPA segmentation, this unsupervised approach focuses on removing muscle and bone from CT labels, enhancing the model's learning of CT features. We improved label accuracy by training the segmentation model with a self-supervised loss function that specifically corrects for muscle and bone, as detailed in Equation (7).

(7) $${\mathrm{Loss}=L}_{\mathrm{DSC}}+w_{m}L_{\mathrm{Mucl}e_{i}nclusion}^{\mathrm{SS}}+w_{b}L_{\mathrm{Bon}e_{i}nclusion}^{\mathrm{SS}}$$

(8) $$L_{\mathrm{DSC}}=1-\frac{\left(Pred\cap GT\right)}{\left(Pred\cup GT\right)}$$

(9) $$L_{\mathrm{Mucl}e_{i}nclusion}^{\mathrm{SS}}=\frac{\left(Pred\cap {\mathrm{CT}}_{\mathrm{Muscle}}\right)}{\left(Pred\right)}$$

(10) $$L_{\mathrm{Bon}e_{i}nclusion}^{\mathrm{SS}}=\frac{\left(Pred\cap {\mathrm{CT}}_{\mathrm{Bone}}\right)}{\left(Pred\right)}$$

$\mathrm{Pred}:ModelPrediction;\;\;GT:PhysicianContour;\;\;{\mathrm{CT}}_{\mathrm{Muscle}}:-29\leq CT\leq 150;\;\;{\mathrm{CT}}_{\mathrm{Bone}}:\;300\leq CT\leq 1200$; $w_{m}=0.1,w_{b}=0.01$

Muscle and bone inclusion loss is calculated between the model prediction and muscle/bone threshold CT. $w_{m}andw_{b}$ were optimized using grid search.

### 2.3 Training details

For training the model we randomly applied data augmentation techniques such as rotating by small angles (<10°), image scaling, and image flipping for more effective learning. Adaptive histogram equalization was used for data preprocessing to enhance edge definitions and improve local contrast for CT, while min–max normalization was used for MRI. The network was trained with Adam optimizer with an initial learning rate of 0.001 with exponential decay. This was implemented in Keras with TensorFlow backend and trained on one 32 GB NVIDIA Tesla V100 GPU. The batch size was four due to memory limitations.

### 2.4 Model performance evaluation

#### 2.4.1 Quantitative evaluation

We performed ablation studies to understand the importance of each unique component of the model and to evaluate their effectiveness. The UNet model with a single encoder, squeeze and excite blocks, and without modality attention served as the baseline model in our study and is denoted as ‘SNet’ in the comparison. The proposed architecture that has separate encoders for MRI, CT, and modality attention is denoted as ‘SNet-MA’ in the comparison. We compared the DSC values of predicted IPAs by using paired two-sided t-tests for the following network architectures:

(1) SNet – baseline model trained with only DSC loss.
(2) SNet with Muscle-Bone loss – baseline model trained with DSC + Muscle-Bone loss.
(3) SNet-MA – proposed architecture but trained with only DSC loss; and.
(4) SNet-MA with Muscle-Bone loss − proposed architecture and trained with DSC + Muscle-Bone loss.

Additionally, we evaluated the effectiveness of modality dropout to allow the flexibility of MRI and/or CT as input for the IPA segmentation. We trained SNet-MA with the Muscle-Bone loss model without and with Modality Dropout (MoDO) and compared the contour quality with CT only as input versus MRI and CT as input. We reported the segmentation results using three quantitative metrics: Dice similarity coefficient score (DSC) as a percentage, average surface distance (ASD) in millimeters, and Hausdorff95 (HD95) in millimeters. Together, these metrics provided a comprehensive quantitative evaluation.

#### 2.4.2 Dose evaluation

We further evaluated the dosimetric accuracy of the AI segmented IPA structure. We took the clinical plan generated with the physician’s IPA contour in the 14 test cases and mapped the planned dose on the AI segmented IPA contour to compare important dose-volume parameters. For the IPA, important dose-volume parameters were defined based upon constraints from our ongoing multi-center randomized trial to be the mean (Dmean) and the volume receiving >=20 Gy dose (V20). These values were compared between manual segmentation and predicted IPA segmentation for the 14 test patients.

#### 2.4.3 Clinical observer study

Since this AI model was developed to assist physicians for contouring the IPA, we hypothesized that the AI model improves contour acceptance when used as “warm start” for inexperienced physicians to contour the IPA. We evaluated our hypothesis by having inexperienced physicians contouring the IPA with and without AI assistance and compared efficiency and quality improvements. The recorded time taken to complete the contour with and without AI assistance was used as the efficiency measure. Next, an expert physician scored the contours predicted by AI (AI-raw), inexperienced MD’s contour without AI (MD-raw), and inexperienced MD’s contour with AI assistance (AI-MD).

Scores from 1 to 4

1. Unacceptable
2. Acceptable with major corrections
3. Acceptable with minor corrections
4. Acceptable as-is

## 3 Results

### 3.1 Quantitative metrics

Our study introduces the SNet with Modality attention (SNet-MA) model, enhanced by Muscle-Bone loss, demonstrating a significant advancement in Internal Pudendal Arteries (IPA) segmentation. This model achieved a Dice Similarity Coefficient (DSC) of 62 %, markedly surpassing other models (p < 0.005). A detailed ablation study, covering 14 test cases, is summarized in Table 1. Notably, the SNet baseline model, when combined with Muscle-Bone and DSC loss, outperformed the use of DSC loss alone. However, SNet-MA without Muscle-Bone loss showed lesser effectiveness, underscoring the importance of muscle and bone features in CT scans.

**Table 1:** Ablation study results for various components of the baseline UNet with squeeze and excite blocks (SNet) and the proposed method (SNet with modality attention, SNet-MA). SNet-MA in conjunction with Muscle-Bone loss function has a DSC accuracy of 62 % and performs significantly (p < 0.005) better than other models.

**Architecture**	**Input**	**Loss**	**DSC (%)**
SNet	2 channel input	DSC loss	56.12 ± 8.12
		DSC loss + Muscle-Bone Loss	57.59 ± 7.95
SNet-MA	2 separate encoders for MRI&CT	DSC loss	54.24 ± 9.50
		DSC loss + Muscle-Bone Loss	61.71 ± 7.00

**Fig. 2:**
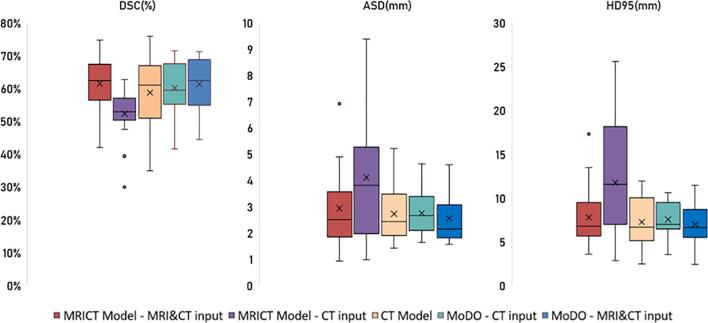
Boxplot showing Dice Similarity Coefficient (DSC), Average Surface Distance (ASD) and Hausdorrf95 Distance (HD95) metric values for 1) MRICT Model with both MRI and CT as inputs; 2) MRICT with only CT as input; 3) CT model trained and tested with only CT data; 4) MoDO (MRICT Model with **Mo**dality **D**rop**o**ut) with both MRI & CT as input; 5) MoDO with only CT as input. Using modality dropout significantly improves the performance when only CT is available compared to a model trained without modality dropout.

Fig. 2 in our analysis visually contrasts various AI models and input image combinations, using metrics like DSC, Average Surface Distance (ASD), and Hausdorff Distance (HD95). The model without modality dropout, 'MRICT', and the one with dropout, 'MoDO', are compared. MRICT, using both CT and MRI, sets the baseline. Its performance drops (Mean DSC 44 %) when only CT is used, in contrast to a CT-only model (Mean DSC 54.1 %). The MoDO strategy addresses this issue effectively, even outperforming the CT-only model when MRI is excluded (DSC 57.2 %). The MoDO model, integrating SNet-MA with both DSC + Muscle-Bone loss and modality dropout, emerges as the most effective, achieving a DSC of 62.2 %, ASD of 2.54 mm, and HD95 of 7 mm, indicating its superiority in accurately segmenting the IPA.

**Fig. 3:**
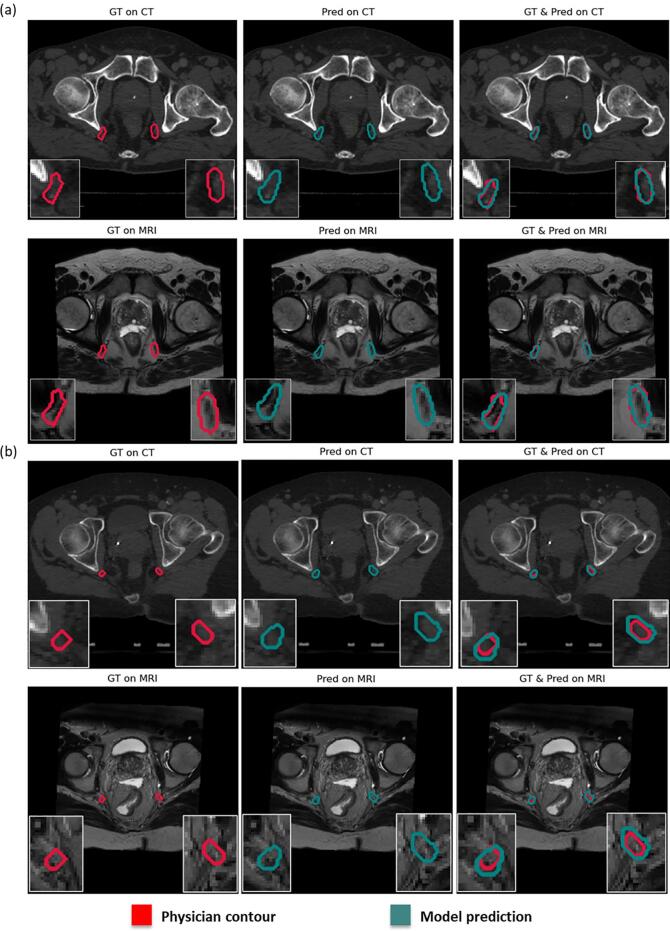
Two examples of predictions vs physician contours. GT: Physician contours, Pred: Model predictions.

Fig. 3 illustrates two example patients with physician and AI segmented IPA contours with CT alone as input versus MRI alone as input. It can be observed that most of the features for segmentation come from MRI. However, AI segmentation with CT alone as input also resulted in satisfactory quality of contour.

### 3.2 Dose evaluation

**Fig. 4:**
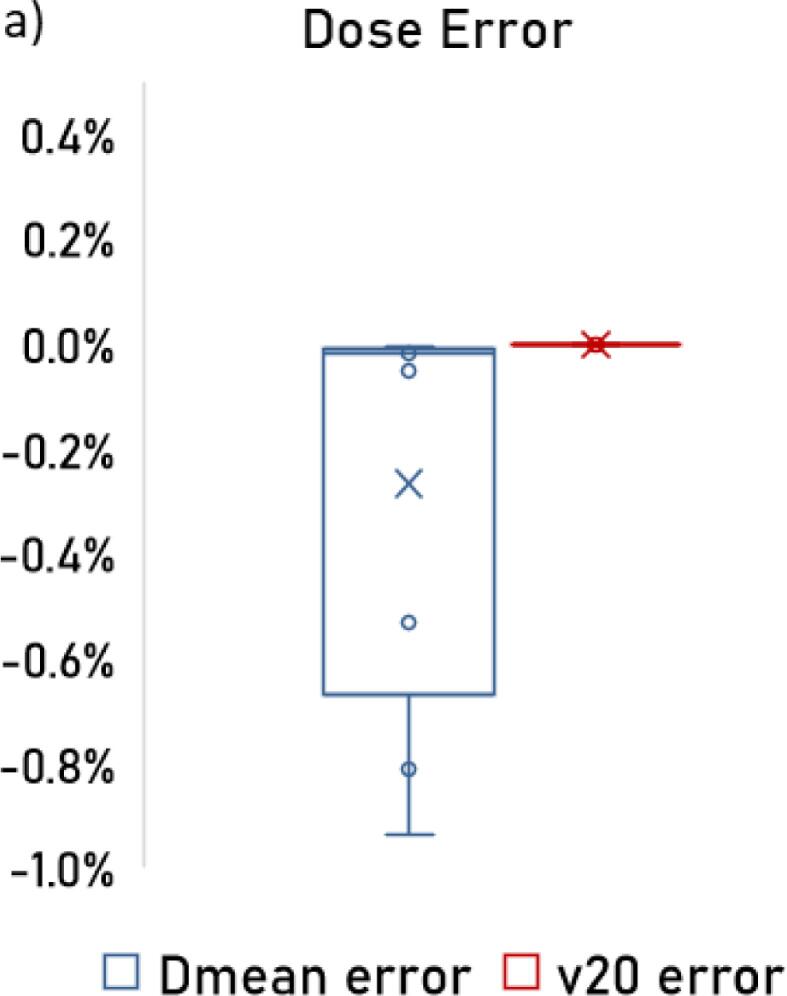
a) Boxplot showing error in Dmean and error in v20 for the model predicted IPA when compared to physician IPA.

Fig. 4 shows the DVH comparison for physician and AI segmented IPA contours for the 14 test cases. Clinical treated plan dose mapped on AI segmented IPA contour demonstrated no difference in V20, and the differences in Dmean were within 1 % and statistically and clinically insignificant.

### 3.3 Clinical observer study

**Fig. 5:**
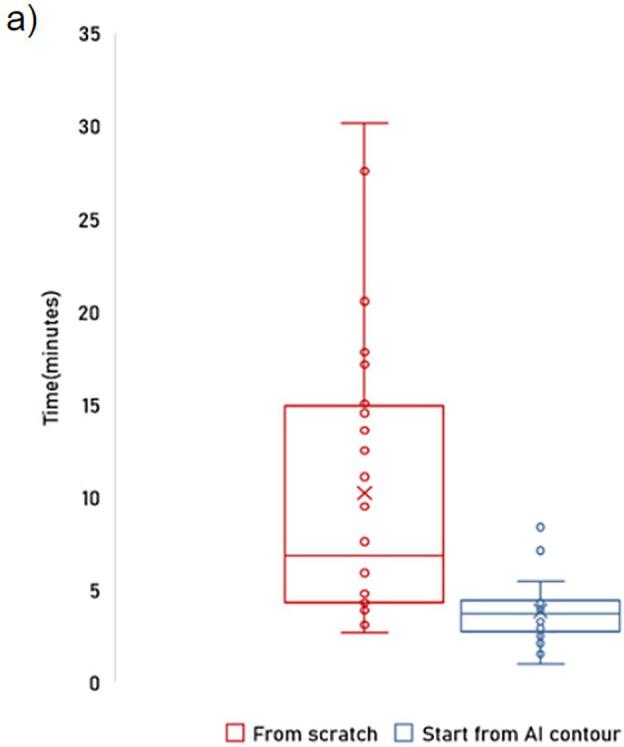
a) Boxplot of time in minutes taken by an inexperienced physician when starting from scratch versus when starting from AI segmented IPA for 30 patients.

Fig. 5a illustrates that inexperienced physicians took significantly less time contouring with AI assistance (4.7 min) compared to starting from scratch (10.8 min). AI-generated contours (AI-raw) averaged a score of 3.7, with 26 out of 30 patients scoring 4. Physician-corrected AI contours (AI-MD) significantly outperformed manually drawn ones (MD-raw), averaging a score of 3.7 with 23 of 30 scoring 4, versus MD-raw’s average of 3.1. In 55 % of cases, an experienced physician deemed corrections by inexperienced physicians unnecessary. In 31 % of cases, these modifications didn't significantly change contour quality, and in 14 % of cases, the experienced physician preferred the physician-corrected contour over the AI-generated one.

## 4 Discussion

Prostate cancer patients face a significant risk of developing radiation-induced erectile dysfunction (RiED), estimated at about 50 %. Research [3] indicates that RiED often results from vascular dysfunction in small arteries, like the Internal Pudendal Artery (IPA). However, in conventional radiation therapy, the IPA is not typically segmented or considered as an organ-at-risk (OAR), which could contribute to the incidence of RiED.

Our study developed a deep CNN for accurate IPA segmentation, focusing on clinical practicality and ease of maintenance. We addressed label variability with a novel loss function for unsupervised error correction and consistent contouring. The model includes a modality attention framework with deformable convolutions, efficiently handling misalignments between MRI and CT images.

One of the pivotal advancements in our approach is the introduction of a modality dropout strategy. This allows the model to maintain effective performance even in the absence of MRI inputs, accommodating cases where MRI is not available. Notably, our model's output is highly correlated with features visible in MRI scans, yet it performs equally well, or even better, when trained with CT data alone. This flexibility was evident when comparing the performance of our model with others trained solely on CT data.

A key part of our research was evaluating the geometric and dosimetric accuracy of the AI-generated contours. These were found to be similar to manual contours created by expert oncologists. Due to the IPA's small size, even minor discrepancies in contouring can significantly affect quality metrics. However, our study faced a limitation: the absence of a defined standard for satisfactory contour quality. To address this, we compared the dosimetric values of AI-generated contours with those delineated by experts. The AI model matched expert oncologists in contour quality and enhanced consistency for inexperienced physicians, as confirmed by an observer study.

Previous research has extensively explored deep learning (DL) models for automated IPA segmentation on MRI [23], which offers superior soft tissue contrast. However, our study aimed to achieve effective IPA segmentation on CT scans, a more challenging medium due to its limited soft tissue contrast. Despite these challenges, our model achieved a median Dice Similarity Coefficient (DSC) of 62 % on CT, compared to 79 % directly on MRI in other studies.

One limitation of our study is the potential lack of generalizability when models are trained with data from a single institution. This underscores the need for future testing on external datasets, should they become available. Another challenge is the rarity of IPA segmentation, resulting in a smaller dataset for training deep learning models. Furthermore, the study relied on a single expert reviewer for contour evaluations and did not validate the relationship between IPA dose constraints and ED outcomes.

Our AI model for IPA segmentation offers multiple clinical uses, such as quality assurance in trials, aiding new physicians in contouring, generating IPA contours for routine clinical planning, and analyzing patient data for outcome correlations. Integrated with systems for nearby pelvic structures, it enhances CT segmentation in radiotherapy, aiming for better treatment and possibly lower RiED rates.

## 5 Ethics approval and consent to participate

Patient data underwent anonymization prior to the initiation of the research, ensuring the information no longer pertained to identifiable individuals. When data no longer retains identifiable associations or has been effectively anonymized to prevent re-identification, it falls outside the scope of data protection law as it ceases to be considered personal data. Consequently, this research, given the anonymized nature of the data, did not necessitate ethical approval from the ethics committee.

## CRediT authorship contribution statement

**Anjali Balagopal:** Writing – original draft, Methodology, Software, Funding acquisition. **Michael Dohopolski:** Writing – review & editing, Methodology. **Young Suk Kwon:** Writing – review & editing. **Steven Montalvo:** Writing – review & editing. **Howard Morgan:** Writing – review & editing. **Ti Bai:** Writing – review & editing, Methodology. **Dan Nguyen:** Writing – review & editing, Methodology, Software. **Xiao Liang:** Writing – review & editing. **Xinran Zhong:** Writing – review & editing. **Mu-Han Lin:** Writing – review & editing, Methodology, Supervision. **Neil Desai:** Conceptualization, Writing – review & editing, Methodology, Supervision. **Steve Jiang:** Conceptualization, Writing – review & editing, Methodology, Supervision, Funding acquisition.

## Declaration of Competing Interest

The authors declare that they have no known competing financial interests or personal relationships that could have appeared to influence the work reported in this paper.
